# Characterizing the Complexity of Weighted Networks via Graph Embedding and Point Pattern Analysis

**DOI:** 10.3390/e22090925

**Published:** 2020-08-23

**Authors:** Shuo Chen, Zhen Zhang, Chen Mo, Qiong Wu, Peter Kochunov, L. Elliot Hong

**Affiliations:** 1 Division of Biostatistics and Bioinformatics, Department of Epidemiology and Public Health, School of Medicine, University of Maryland, Baltimore, MD 21201, USA; 2 Maryland Psychiatric Research Center, Department of Psychiatry, School of Medicine, University of Maryland, Baltimore, MD 21228, USA; chen.mo@som.umaryland.edu (C.M.); PKochunov@som.umaryland.edu (P.K.); Ehong@som.umaryland.edu (L.E.H.); 3 Department of Accounting, College of Business and Economics, Towson University, Towson, MD 21252, USA; zzhang@towson.edu; 4 Department of Mathematics, University of Maryland, College Park, MD 20742, USA; qwu1221@terpmail.umd.edu

**Keywords:** brain network, entropy, graph embedding, point process, schizophrenia, weighted network

## Abstract

We propose a new metric to characterize the complexity of weighted complex networks. Weighted complex networks represent a highly organized interactive process, for example, co-varying returns between stocks (financial networks) and coordination between brain regions (brain connectivity networks). Although network entropy methods have been developed for binary networks, the measurement of non-randomness and complexity for large weighted networks remains challenging. We develop a new analytical framework to measure the complexity of a weighted network via graph embedding and point pattern analysis techniques in order to address this unmet need. We first perform graph embedding to project all nodes of the weighted adjacency matrix to a low dimensional vector space. Next, we analyze the point distribution pattern in the projected space, and measure its deviation from the complete spatial randomness. We evaluate our method via extensive simulation studies and find that our method can sensitively detect the difference of complexity and is robust to noise. Last, we apply the approach to a functional magnetic resonance imaging study and compare the complexity metrics of functional brain connectivity networks from 124 patients with schizophrenia and 103 healthy controls. The results show that the brain circuitry is more organized in healthy controls than schizophrenic patients for male subjects while the difference is minimal in female subjects. These findings are well aligned with the established sex difference in schizophrenia.

## 1. Introduction

The research of complex networks has attracted significant attention in the last few decades. Complex networks are a natural representation of real-world interactive processes among multiple units [1]. For example, social, financial, gene-regulation, and brain networks are complex networks, which are neither purely random nor regular [2,3,4,5]. Complex networks consist of organized (although often latent) network topological structures, and they exhibit properties such as scale-free and small-worldness [6]. Analytical models have become fundamental tools to characterize the complex structure and intrinsic mechanisms of complex networks [7,8].

The quantification of the intrinsic network complexity of complex networks is a fundamental problem in network analysis. The complexity, as a permutation invariant graph characteristic, has proved to be more effective than a number of simple alternatives, including edge density, perimeter, and path length. Recently, advanced computational models have been developed to calculate the von Neumann entropy and Shannon entropy of an unweighted graph/network [9,10,11]. The network entropy provides a robust measurement of the complexity of a weighted network/graph. The network entropy can also be used as a graph descriptive statistic and applied to group-level network analysis, for example, to compare the complexities between networks that were collected from two cohorts of subjects: a group of patients with brain diseases and a group of healthy controls [12].

**Figure 1:**
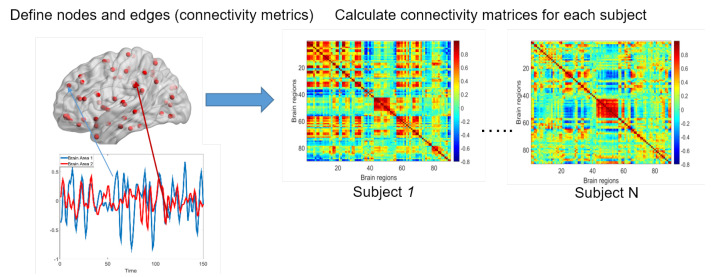
Functional brain connectivity can be denoted by a weighted network, where each node is a brain area and the continuous edge weight represents the connection strength between a pair of brain locations (e.g., calculated by the Fisher’s Z transformed Pearson correlation coefficient between the two blood-oxygen-level-dependent (BOLD) time sequences).

The brain network analysis has become an active research area in the last decade [13]. The recent development in neuroimaging technology has facilitated the non-invasive measurement of brain circuitry. For example, diffusion tensor imaging (DTI) and tractography can be used to construct white matter structural networks, while the coactivation patterns of blood-oxygen-level-dependent (BOLD) signals from functional magnetic resonance imaging (fMRI) can be calculated in order to estimate functional brain networks [14]. The human brain network is a complex network, where brain areas are denoted by graph nodes, and the structural/functional connections are represented by edges [4]. The brain network consists of complex, organized, yet latent topological structures and exhibits network properties, including scale-free, small-worldness, and high-modularity [3]. These network characteristics have been associated with brain diseases and behaviors. In these studies, brain imaging data are acquired for each individual to compute the brain network, and then network complexity scores can be calculated for all participants to perform group-wise statistical analyses (see Figure 1).

The network entropy-based complexity measurement is undoubtedly critical and informative because the complex human cognitive functions and behaviors are associated with organized brain networks [15]. However, the calculation of complexity of brain networks has been challenging because (i) edge weights in the brain network are continuous variables following unknown distributions (e.g., an infinite mixture model of multivariate distributions with unknown covariance); (ii) the empirical distributions of edges can be misleading due to the substantial noise of edge; and, (iii) edges are correlated with each with a large and unknown covariance structure constrained by network and spatial structures. Moreover, simply binarizing weighted edges into binary edges by an arbitrary threshold is subject to massive information loss and reduced statistical power. A straightforward alternative approach to measure the complexity and non-randomness of a weighted network is to directly extract the network topological structure from the weighted adjacency matrix. However, the false positive and false negative noise in the edge weights can easily distort the recovery of the true underlying topological structure. This is more challenging when the structure is complicated, such as a mixture of k-partite and rich-club [16]. To address this unmet need, we propose a new computational method to measure the complexity via graph embedding and statistical point pattern analysis. We first project each weighted complex network into a low dimensional vector space and perform point pattern analysis via the statistical point process. The distribution of points from an unorganized Erdős Renyi random weighted graph is distinct from an organized brain network, which can be reflected by our complexity measurement. Note that our method does not require learning the distribution of edges in the weighted adjacency matrix nor the underlying topological structure, thus it is computationally convenient and is robust to false positive noise.

The rest of this paper is organized, as follows. In Section 2, we introduce the definition of the metric of complexity for weighted complex network. Section 3 describes the validation of the proposed approach using various simulated data and the results of an application to schizophrenia functional network analysis. The results, for the first time, reveal that the complexity of brain networks significantly differs between male healthy controls and schizophrenic patients, whereas they are similar in females, which may further explain the well-established difference between sex in schizophrenia.

## 2. Methods

### 2.1. Background

The graph notation G={V,E,W} is often used to denote the weighted network, where *V* is the set of nodes/vertices, *E* is the edge set (links between nodes). We use a weighted adjacency matrix $W_{n\times n}$ to denote the edge weights, where an entry $w_{ij}$ is a real number and represents the connectivity strength between the two nodes i,j,0<j<i<n. For example, the correlation coefficient can be used and $-1\leq w_{ij}\leq 1$.

The network entropy has been well established for binary networks that are based on a Bernoulli distribution model [9,11]. However, the network entropy for the weighted network is challenging because the edge weights in ***W*** a mixture multivariate normal distributions with a large and unknown covariance matrix (the number of parameters is at the order of $n^{4}$) and unknown mixture component [17]. The parameter estimation is often intractable, and the computation of entropy is then challenging.

In the current research, we are facing a more challenging task, because our data are a group of weighted networks. For a group-level brain connectivity analysis, we denote the data $\left\{W_{n\times n}^{1},W_{n\times n}^{2},\cdots ,W_{n\times n}^{S}\right\}$. For one study subject, we have a weighted network $W_{n\times n}^{s}$, s=1,⋯,S, and a vector of clinical covariates $Z^{s}$ (e.g., clinical status, age, and sex). The complexity of $W_{n\times n}^{s}$ can represent the level of brain organization and efficiency, which influences the symptoms of brain disorders (e.g., cognitive deficit).

We develop a novel framework to quantify the complexity and non-randomness based on graph embedding and point pattern analysis to overcome the computational challenge and understand the association between the organization of brain connectivity networks and the clinical symptoms of mental disorders.

### 2.2. Graph Embedding of a Weighted Network

**Figure 2:**
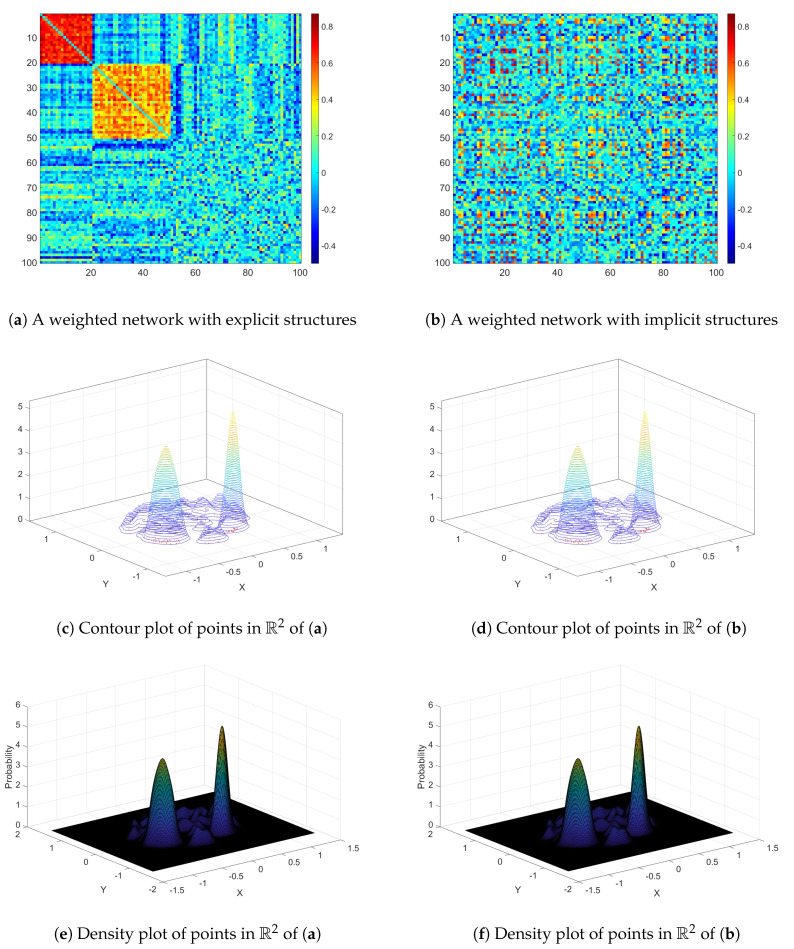
The demonstration of permutation invariance for points (nodes) in a low dimensional vector space $R^{k}$ via graph embedding. (**a**,**b**) are the weighted adjacency matrix for two isomorphic weighted graphs, in that, (**b**) can be transformed to (**a**) by rearranging the order of nodes. Nodes in (**a**,**b**) are projected into $R^{k}$ (*k* = 2 for demonstration). The distribution pattern of nodes in $R^{k}$ for the two weighted networks. (**c**,**d**) are contour plots, and (**e**,**f**) are density plots, which all show very similar point patterns of two weighted networks. Therefore, the point pattern in $R^{k}$ can reflect the intrinsic complexity and is permutation invariant.

Graph embedding projects a graph into a low dimensional vector space $R^{k}$ while preserving the graph information and facilitates the efficient computation of graph analytics [18]. A key advantage of graph embedding in our application is its invariance to the isomorphic mapping of the graph: the projection of our weighed network in a low dimensional vector space remains unchanged when the order of nodes shuffles (see Figure 2). This property alleviates the challenging task of extracting complicated and unknown topological structure from the weighted graph ***W***, because the distribution of points in $R^{k}$ is independent of the detection of graph topological structure (e.g., allocating points to clusters). The complexity and non-randomness of the original weighted network can be well captured by the points in $R^{k}$.

Graph embedding maps the weighed network W:N×N→R and a set of nodes $V=\{v_{1},v_{2},\cdots ,v_{n}\}$ into vectors ${\tilde{X}}_{k\times n}=\left\{X_{1},X_{2},\cdots ,X_{n}\right\}$ in $R^{k}$, such that

(1) $$\begin{array}{c} \underset{\tilde{X}}{arg min}\left|\right|{\tilde{X}}^{T}\tilde{X}-\frac{1}{2}\mathrm{JWJ}{\left|\right|}_{F}, \end{array}$$

where $J=I_{n}-1/n1_{n}1_{n}^{t}$ and $I_{n}$ and $1_{n}$ are the identity matrix and a vector of ones respectively. ${\tilde{X}}_{k\times n}$ provide a set of *k*-dimensional coordinates for the nodes $V=\{v_{1},v_{2},\cdots ,v_{n}\}$. Next, we apply spatial statistical methods to analyze the pattern of points in the $R^{k}$ space and measure the complexity and non-randomness of the connectivity that is based on the point distribution patterns.

### 2.3. Point Pattern Analysis in the $R^{k}$ Space

**Figure 3:**
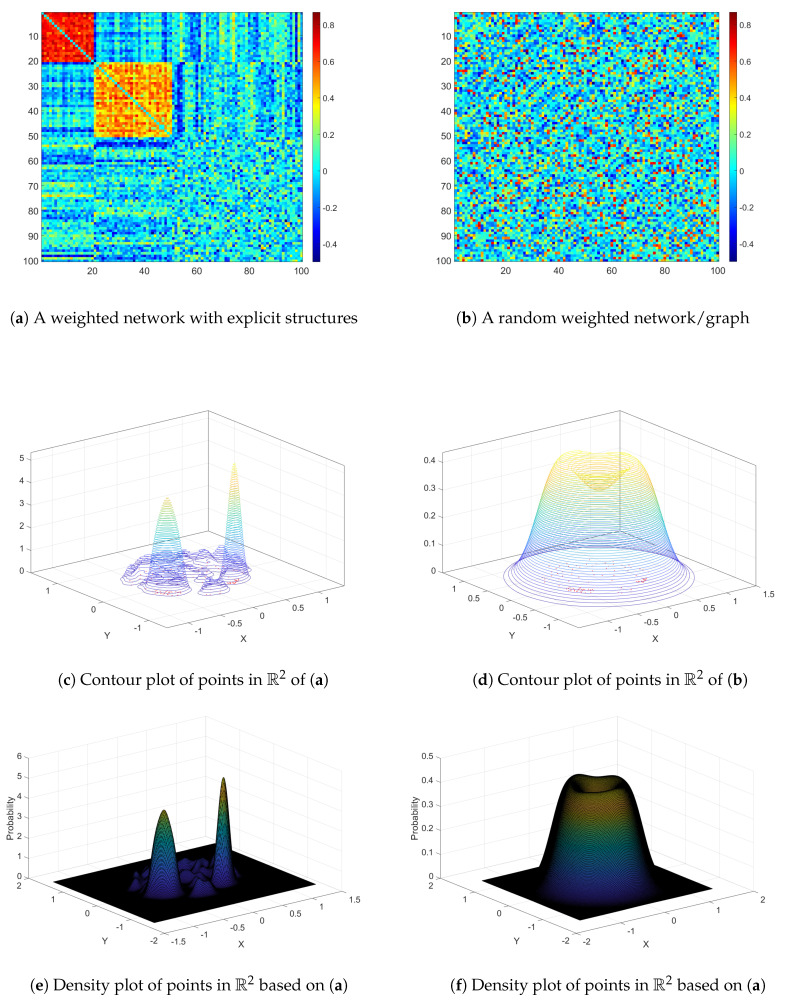
The demonstration of point patterns in a low dimensional vector space $R^{k}$ for an organized graph (**a**) vs. a random graph (**b**) with an identical edge weight distribution. Nodes in (**a**,**b**) are projected into $R^{k}$ (*k* = 2 for demonstration). The distribution pattern of nodes in $R^{k}$ for the two weighted networks. (**c**,**d**) are contour plots, and (**e**,**f**) are density plots. Plots (**c**–**f**) show distinct point patterns between the weighted network with an organized structure and a random weighted graph. The difference can be quantified by a cross entropy measurement.

In the $R^{k}$ space, each point represents a node in the weighted network. The distribution patterns of the points in $R^{k}$ reflect their connectivity in the weighted adjacency matrix. For example, the points that are mapped from a random weighted graph show a point pattern of complete spatial randomness (CSR). In contrast, points mapped from a weighted network with an organized topological structure show a clustered pattern (see Figure 3).

In spatial statistics, stochastic point processes, for example, the Poisson process and Cox process, are often used to model the point patterns [19]. In that, the number of nodes located within a radius of *r* centered at a point/node *i* follows a Poisson distribution. Under the assumption of a random graph (i.e., CSR in $R^{k}$), the point process can be modeled as a homogeneous point process with the density λ. Hence, we can use the cross entropy (CE) between the observed point pattern from an organized weighted network and the point process by the random graph to characterize the complexity of a weighted network. Specifically, the cross entropy is

(2) $$L\left(r\right)=-\frac{1}{n}\sum_{i=1}^{n}\sum_{j\neq i}\left\{I\left(d_{ij}<r\right)\right\}\log\left(\pi r^{2}\lambda \right),$$

where *r* is the radius, $d_{ij}$ denotes the Euclidean distance between points *i* and *j*, λ is density parameter, and $\pi r^{2}\lambda$ is the expected number of points within an area centered at point *i* and radius *r*. In practice, we estimate L(r) similarly to the estimate of Ripley’s *K* function since L(r)=cK(r) (*c* is a constant). The Poisson distribution for the nodes at the boundary of the point distributed area can be distorted because of the edge effect [20]. The distribution function presented in Figure 3d is example of the boundary/edge effect. Thus, the boundary/edge effect can be corrected, which also has been well developed by the Ripley’s *K* function estimation. Specifically, the *K* function is estimated by

(3) $$\hat{K}\left(r\right)=\lambda^{-1}\frac{1}{n}\sum_{i=1}^{n}\sum_{j\neq i}\eta_{ij}\left\{I\left(d_{ij}<r\right)\right\},$$

where the weight function $\eta_{ij}$ provides the edge correction. Then, we can conveniently calculate $\hat{L}\left(r\right)=c^{-1}\hat{K}\left(r\right)$. Last, we integrate the measure by *r* and name the measurement as **g**raph **em**bedding based point process cross **e**ntropy (*Geme* ).

(4) $$\hat{\mathrm{Geme}}=\int \hat{L}\left(r\right)dr,$$

*Geme* is built on the contrast between the observed point pattern of the weighted network vs. the point pattern of CSR (projected from a non-organized weighted network random graph) in the $R^{k}$ space. In the low dimensional space $R^{k}$, the complexity of weighted networks is well captured by the point/node distribution patterns because the Euclidean distance between nodes can appropriately represent the edge weights in the weighted adjacency matrix. The point patterns hence reflect the complexity of weighted networks. The point process methods developed in spatial statistics then provide a convenient pathway to characterize the ‘complexity’ of the point patterns. The computational complexity of *Geme* is $O(n^{2.367})$, and thus *Geme* can be scalable to large networks. In summary, *Geme* is a new metric to measure the complexity and non-randomness of a weighted network and graph. *Geme* can avoid the arbitrary cut-off to binarize a weighted graph to a binary graph and is invariant to all isomorphic forms of a weighted graph and can quantify the complexity robustly.

## 3. Results

### 3.1. Simulations

We first validate and evaluate the proposed complexity metric *Geme* using synthetic data sets. In the current research, we focus on group level comparison and test whether *Geme* can accurately capture the difference of complexity between groups of networks with different topological structures. We illustrate examples using the commonly used two group setting; however, the conclusion can be easily extended to the regression setting. Specifically, we simulate a set of weighted networks (i.e., brain networks for multiple subjects) from two covariance matrices $\Sigma_{n\times n}^{0}\neq \Sigma_{n\times n}^{1}$, where *n* is the number of nodes. For subjects $s=1,2,\cdots ,S_{0}$ in group one, we simulate $Z_{n\times T}^{s}∼N\left(\mu_{0},\Sigma_{n\times n}^{0}\right)$ and $Z_{n\times T}^{s^{\prime }}∼N\left(\mu_{1},\Sigma_{n\times n}^{1}\right)$ with $s^{\prime }=S_{0}+1,S_{0}+2,\cdots ,S_{0}+S_{1}$. *T* is the number of volumes of the image, for example, the number of time points in fMRI data. In this application, we let $S_{0}=S_{1}=100$. For the sake of simplicity, we let $\mu_{0}=\mu_{1}=0$. For a subject *s*, we can calculate the weighted network by $W_{n\times n}^{s}=diag{\left(U\right)}^{1/2}\;U\;diag{\left(U\right)}^{1/2},U=\frac{1}{T-1}Z^{s}{\left(Z^{s}\right)}^{T}$ for each subject. As a result, we obtain a set of weighted adjacency matrix $\left\{W^{1},\cdots ,W^{S_{0}},W^{S_{1}+1},\cdots ,W^{S_{1}}\right\}$. We set the two covariance matrices $\Sigma^{0}$ and $\Sigma^{1}$ as

$$\Sigma^{0}=\left[\begin{array}{cccc} I_{a_{1}}\left(1-\rho_{1}\right)+J_{a_{1}}\rho_{1} & & & \\ & I_{a_{2}}\left(1-\rho_{2}\right)+J_{a_{1}}\rho_{2} & & \\ & & \ddots & \\ & & & 1 \end{array}\right],\;\;\Sigma^{1}=\left[\begin{array}{cccc} I_{b_{1}}\left(1-\zeta_{1}\right)+J_{b_{1}}\zeta_{1} & & & \\ & I_{b_{2}}\left(1-\zeta_{2}\right)+J_{b_{1}}\zeta_{2} & & \\ & & \ddots & \\ & & & 1 \end{array}\right].\;$$

Both matrices exhibit a community network structure, where $\left\{a_{1},a_{2}\right\}$ and $\left\{b_{1},b_{2}\right\}$ are sizes of community subnetworks and $\left\{\rho_{1},\rho_{2}\right\}$ and $\left\{\zeta_{1},\zeta_{2}\right\}$ are correlations between nodes in the communities. The off-diagonal blocks are zeros. We simulate data using different settings by set various values of $\left\{a_{1},a_{2}\right\}$ and $\left\{b_{1},b_{2}\right\}$, and $\left\{\rho_{1},\rho_{2}\right\}$ and $\left\{\zeta_{1},\zeta_{2}\right\}$. The larger sizes of communities indicate a greater complexity of the weighted network because it is more organized. We consider the variation of correlations as noises because point pattern in the low dimensional space is more clear when $\left\{\rho_{1},\rho_{2}\right\}$ and $\left\{\zeta_{1},\zeta_{2}\right\}$ are larger and more likely to distinguish from false positive edges. Because we have two matrices, we set $\Sigma^{0}$ as fixed and vary the parameters in $\Sigma^{0}$. We let $\left\{a_{1},a_{2}\right\}=\left\{20,30\right\}$ and $\left\{b_{1},b_{2}\right\}=\left\{20,30\right\},\left\{20,20\right\},\left\{10,15\right\}$. We set $\Sigma^{0}$ as a fixed matrix with $\left\{\rho_{1},\rho_{2}\right\}=\left\{0.5,0.6\right\}$ and vary the parameters in $\Sigma^{1}$ by letting $\left\{\zeta_{1},\zeta_{2}\right\}=\left\{0.5,0.6\right\},\left\{0.4,0.5\right\},\left\{0.3,0.4\right\},\left\{0.2,0.3\right\}$. For each setting, we simulate $\left\{Z_{s}\right\}$ for 100 times, calculate *Geme* for all subjects, and then test whether the difference of complexity of weighted networks (*Geme*) can be detected between the two groups. In the Method section, we demonstrate one example subject with the parameters of $\left\{\rho_{1},\rho_{2}\right\}=\left\{0.6,0.5\right\}$ and $\left\{a_{1},a_{2}\right\}=\left\{20,30\right\}$ in Figure 2 and Figure 3. The average computational time for a subject with n=100 nodes is 0.11 s on a PC with a CPU i7 3.6G HZ and 64G ROM.

The results presented in Table 1 show that *Geme* can sensitively reflect the complexity of weighted networks. When the difference of complexity between the two groups of weighted networks is small (e.g., one subnetwork size in group one is larger than group two by ten nodes), we have the power of 73%±3.2% to detect the difference of complexity between the two groups of weighted networks with a statistical significance α=0.05, when $\left(\zeta_{1},\zeta_{2}\right)=\left(\rho_{1},\rho_{2}\right)$. The power is reduced when $\left(\zeta_{1},\zeta_{2}\right)<\left(\rho_{1},\rho_{2}\right)$ and connected edges are less different from unconnected edges. We consider the signal to noise ratio (SNR) in a weighted network is lower when $\left(\zeta_{1},\zeta_{2}\right)$ is closer to zero. When the difference of complexity is larger (i.e., the subnetwork size difference is larger), we are more likely to detect the difference using *Geme*. When the subnetwork difference is about 20, we have a power of 100%. In addition, the statistical power is related to the sample size, and we use a sample size that is close to our motivation data set.

### 3.2. Application to Brain Network Analysis for Schizophrenia Research

We apply the proposed new metric to a resting state fMRI data that were collected from 103 patients with schizophrenia (SZ) and 124 healthy controls (HC). This average age of patients with schizophrenia is 36.88 ± 14.17, and 33.75 ± 14.22 for healthy controls. There are 62 males and 41 females in the SZ cohort and 61 males and 63 females in the HC cohort. There are no systematic differences in age ( *p* = 0.10) or gender (*p* = 0.13) between the two groups. We would refer readers to Adhikari et al. [21] for the details of imaging acquisition and preprocessing procedure. We denote the nodes of a network based on a brain connectivity-based atlas that parcellates the brain into 246 regions of interest (ROIs) (see http://atlas.brainnetome.org/ and [22]). The functional connection (edge weight) between a pair of nodes for each subject is calculated by the covariation between averaged time series from the two corresponding brain ROIs. The Fisher’s Z transformed Pearson correlation coefficient is then used as the edge connection strength and, thus, a group of weighted networks $\left\{W_{246\times 246}^{s}\right\}$. Our goal is to examine whether the complexity of weighted networks is different between SZ and HC.

**Figure 4:**
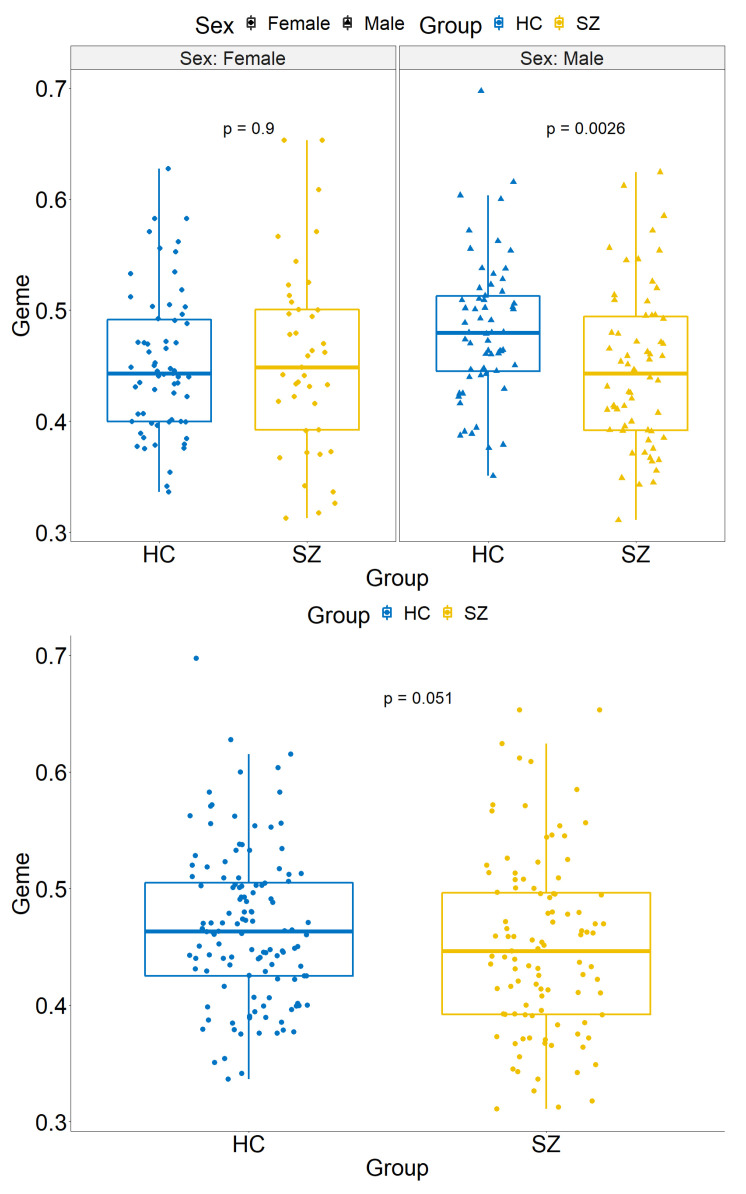
The results show that the weighted network complexity metric *Geme* is lower in patients with schizophrenia than healthy controls for male subjects, whereas the difference is minimal for female subjects. Thus, the brain is less organized in male patients when comparing to male healthy controls while the brain is organized at a similar level in female patients and female healthy controls. The sex moderated difference in brain organization level between SZ and HC may explain the commonly observed phenomenon that male patients have more severe clinical symptoms than female patients. The figure above demonstrates the subgroup analysis for the female and male cohorts, and the figure below shows the results for all subjects.

We first map each weighted network to a low dimensional space $R^{k}$ via graph embedding and then calculate the *Geme* metric for each subject. Further, we perform regression analysis to examine the association between disease and network complexity while adjusting for age and sex. We use a step-wise regression model selection procedure to determine the optimal model regarding the interaction terms. In the final model, the sex and disease interaction term is included. The results show that the interaction term is significant (p=0.032). The network complexity difference between HC and SZ in males is significant (p=0.0026), whereas the group difference not significant in females (p=0.90) based on the stratified analysis. Figure 4 shows the group difference in males and females.

## 4. Discussion

We have developed a new metric *Geme* to characterize the complexity of the weighted complex network. This current research is motivated by a group-level brain network study, which includes a set of weighted brain networks with different clinical statuses. We aim to investigate whether brain disorder can be associated with the complexity of the functional brain networks. The weighted network is different from the binary network, as the values of edges are continuous variables. Unlike binary edges, the distribution of continuous edge variables is unknown because it can be a mixture distribution with infinite unknown components. Thus, the existing network entropy metrics are not applicable. On the other hand, the extraction of network topological structure from the weighted network can be difficult due to the distortion of false positive and negative noise. To overcome these challenges, we implement an integrative procedure of graph embedding and statistical point pattern analysis that is invariant to the permutation of the network. Interestingly, we find that the point distribution pattern of points projected from the nodes of weighted networks in the low dimensional space can reflect the complexity of weighted networks. We utilize the statistical techniques for point process analysis to implement a cross entropy metric in order to capture the deviation of point patterns in complex networks from random graph networks. We propose a computationally efficient method to calculate the metric. We perform extensive simulation analysis to validate the new metric. The results show that our metric can sensitively detect the difference in network complexity based weighted network data. We also note that this method is rust to noise.

We further apply the new metric to our data example. In this study, brain imaging data and weighted brain networks were acquired for each of the 124 patients with SZ and 103 healthy controls. We calculate the network complexity metric for each subject and perform statistical analysis. The results reveal new neuropathological findings that the complexity of functional brain networks is significantly higher for HC than SZ in male subjects, while the difference is not significant in females. The sex difference in schizophrenia has been long established [23,24]. Many studies have reported that onset time of females are later than males [25,26]. In addition, the symptoms of female patients with SZ are milder than males [27,28,29]. These external phenotypes can be well reflected by the organization and the complexity of functional connectivity networks [30]. Our findings may provide a viable neurological explanation for the long-established sex difference in schizophrenia. The functional brain networks are more disturbed and less organized in male patients than female patients when comparing to healthy controls, which is associated with more severe symptoms regarding cognitive functions, anhedonia, and social functioning in male patients. We can further the location-specific edges and nodes that cause the complexity difference using recently developed network methods [16,17,31].

In the data example, we show the application of *Geme* to a functional brain connectivity study. In general, *Geme* can be applied to any weighted (functional and structural) network with a positive semidefinite weighted adjacency matrix. In white matter structural network analysis, we find that clinical symptoms (e.g., cognitive functions) are related to the SC connection strengths (e.g., fractional anisotropy—FA levels) instead of the network complexity [32]. Therefore, these SC connection differences cannot be detected *Geme*. In contrast, the complexity of functional networks is more informative than the absolute connectivity strengths because the connectivity scale can be influenced by procedures, like global regression. Thus, *Geme* is more suited to identify the difference of network organization patterns for functional brain network analysis.

In summary, *Geme* is a computationally efficient metric for measuring the complexity of weighted networks. *Geme* can become a complement to the existing von Neumann network entropy metrics [11]. The graph embedding and point pattern analysis strategy may also provide an alternative pathway for network entropy analysis in addition to the exiting spectral entropy methods.

## Figures and Tables

**Table 1:** Simulation results show that *Geme* can reflect the complexity of weighted networks. We summarize the mean and standard error of the chance to reject the null hypothesis (no difference of network complexity between two groups) based on 100 data sets.

	Subnetwork Sizes				
$\left(\zeta_{1},\zeta_{2}\right)$	**(20, 30)**	**(20, 20)**	**(20, 15)**	**(10, 15)**	**(10,10)**
(0.6, 0.5)	0% ± 0%	73% ± 3.2%	100% ± 0%	100% ± 0%	100% ± 0%
(0.4, 0.5)	2% ± 1%	53% ± 3.5%	92% ± 1.9%	100% ± 0%	100% ± 0%
(0.4, 0.3)	5% ± 1.5%	46% ± 3.5%	90% ± 2.1%	100% ± 0%	100% ± 0%
(0.3, 0.2)	9% ± 2%	43% ± 3.5%	91% ± 2%	96% ± 1.4 %	100% ± 0%
